# Adding new childhood vaccines to China's National Immunization Program: evidence, benefits, and priorities

**DOI:** 10.1016/S2468-2667(23)00248-7

**Published:** 2023-11-22

**Authors:** Haijun Zhang, Xiaozhen Lai, Bryan N Patenaude, Mark Jit, Hai Fang

**Affiliations:** a Department of Health Policy and Management, School of Public Health, Peking University, Beijing, China; b International Vaccine Access Center, Bloomberg School of Public Health, Johns Hopkins University, Baltimore, MD, USA; c Department of International Health, Bloomberg School of Public Health, Johns Hopkins University, Baltimore, MD, USA; d Department of Immunization, Vaccines and Biologicals, WHO, Geneva, Switzerland; e Health Economics Research Centre, Nuffield Department of Population Health, University of Oxford, Oxford, UK; f Department of Infectious Disease Epidemiology, Faculty of Epidemiology and Population Health, and Centre for Mathematical Modelling of Infectious Diseases, London School of Hygiene and Tropical Medicine, London, UK; g School of Public Health, University of Hong Kong, Hong Kong Special Administrative Region, China; h China Center for Health Development Studies, Peking University, Beijing, China; i Peking University Health Science Center—Chinese Center for Disease Control and Prevention Joint Research Center for Vaccine Economics, Peking University, Beijing, China

## Abstract

China's National Immunization Program has made remarkable achievements but does not include several important childhood vaccines that are readily available in the private market, such as pneumococcal conjugate vaccine (PCV), rotavirus vaccine, *Haemophilus influenzae* serotype b (Hib) vaccine, and varicella vaccine. We reviewed the literature to assess these four non-National Immunization Program vaccines in terms of their disease burdens, coverage, inequalities, and cost-effectiveness in China and aimed to recommend priorities for introducing them to the National Immunization Program. Based on our calculations using the available evidence, incorporating these vaccines into China's National Immunization Program in 2019 could have averted 11 761 deaths among children younger than 5 years, accounting for 10·29% of the total deaths in children younger than 5 years and reducing the mortality rate from 7·8 per 1000 to 7·0 per 1000. The review showed that 13-valent PCV (PCV13) had the lowest and most inequitable coverage but could prevent the highest number of deaths. In a budgetary analysis for the cohort of newborns in 2023, we estimated that the projected aggregate government costs were US$1954·92 million for PCV13, $1273·13 million for pentavalent rotavirus vaccine, $415·30 million for Hib vaccine, and $221·64 million for varicella vaccine. Our overall multicriteria decision analysis suggested the following priority order for introducing these four non-programme vaccines to the National Immunization Program to benefit the Chinese population: PCV13, rotavirus vaccine, Hib vaccine, and varicella vaccine.

## Introduction

China has substantially reduced the burden of many vaccine-preventable diseases through its National Immunization Program.^1^ The global Expanded Program on Immunization was initiated by the 27th World Health Assembly in 1974.^2^ Subsequently, China established its own national programme on immunisation in 1978, encompassing four routine childhood vaccines against six diseases: BCG, diphtheria-tetanus-pertussis (DTP) vaccine, polio vaccine, and measles vaccine.^3^

Since the establishment of the Expanded Program on Immunization in 1974, it has facilitated the development and licensing of many new vaccines worldwide. Notably, in 2002, China fully integrated the hepatitis B virus (HBV) vaccine into its Expanded Program on Immunization,^4^, ^5^ which was then reorganised and renamed as the National Immunization Program. In 2008, China further expanded the programme to include vaccines for hepatitis A virus (HAV), Measles-Mumps-Rubella (MMR), Japanese encephalitis, and meningitis caused by meningococcal groups A and C. In 2023, China's National Immunization Program includes 13 routine vaccines. Additionally, targeted vaccinations against hantavirus (which causes haemorrhagic fever with renal syndrome), anthrax, and leptospirosis have been made available in epidemic areas or selected populations. China's efforts have resulted in high rates of vaccine coverage that have exceeded 95% of the national and provincial levels since 2016,^6^ and inequalities in access to National Immunization Program vaccines have been minimised as every child is entitled to receive all routine programme vaccines for free. In China, the central government is responsible for procuring and funding National Immunization Program vaccines, whereas local governments fund immunisation services.

However, China's National Immunization Program does not include most of the recently developed vaccines, including four childhood vaccines: pneumococcal vaccine, rotavirus vaccine, *Haemophilus influenzae* serotype b (Hib) vaccine, and varicella vaccine. Among them, pneumococcal, rotavirus, and Hib vaccines have been recommended by WHO for routine immunisations in all regions, and varicella vaccine has been widely used in many countries, such as Australia, Germany, Japan, and the USA.^7^, ^8^, ^9^, ^10^ Although these four childhood vaccines are readily available in China as non-National Immunization Program vaccines, families have to pay for them privately. Including some of these childhood vaccines in the National Immunization Program could improve the programme's effectiveness and sustainability.^1^ However, no new vaccines have been added to the programme since 2008. Consequently, coverage rates for non-programme vaccines in China remain low and substantial inequalities exist among low-income populations and their ability to access these vaccines.^6^ Notably, most of these non-programme vaccines could be domestically produced in China, ensuring a stable and secure supply chain.

The number of doses of National Immunization Program vaccines delivered in China has been declining since 2016 due to a sharp decrease in the size of birth cohorts.^11^, ^12^ Meanwhile, the number of doses of non-programme vaccines approved by the pharmaceutical administrative authority in China has been rising due to an increasing private demand. According to China's National Institutes for Food and Drug Control,^13^ the total number of doses of 13-valent pneumococcal conjugate vaccine (PCV13), rotavirus vaccine, Hib vaccine, and varicella vaccine approved in China increased from 36·01 million in 2016 to 73·08 million in 2020. Non-programme combined vaccines are also used as substitutes for vaccines included in China's National Immunization Program. For instance, the combined vaccine of Diphtheria, tetanus, and acellular pertussis (DTaP), inactivated polio (IPV), and Hib is used as a substitute for the National Immunization Program vaccines of DTaP vaccine, IPV vaccine, oral polio vaccine, and Hib vaccine. The four-valent (ACWY) meningococcal conjugate vaccine, as a non-programme vaccine in China, can substitute the two-valent (AC) meningococcal polysaccharide vaccine in the National Immunization Program. As a result, children have received fewer vaccination doses with the administration of combined non-programme vaccines than with single types of programme vaccines.^14^ In this case, parents choose to substitute single or polysaccharide vaccines for self-funded, non-programme, combined or conjugate vaccines because of convenience or improved protection. If every newborn was immunised with all programme vaccines, the estimated number of doses of these vaccines administered should have been at least 285·63 million in 2022. However, the actual number of doses of programme vaccines procured by the central government was only 223·59 million in 2022, with many doses substituted by non-programme vaccines.^15^ The total procurement cost of National Immunization Program vaccines in the same year was US$390 million ($1=CN¥6·73 in 2022), which accounted for only 0·03% of the total health expenditures in China.^16^ Considering the large population and total health expenditures in China, the governmental budget for National Immunization Program vaccine procurement is substantially low.

In 2020, the World Health Assembly endorsed the Immunization Agenda 2030 (IA2030), a global strategy that aims to achieve a world in which everyone, of all ages, fully benefits from vaccines to improve health and wellbeing.^17^ IA2030 emphasises the principle of leaving no one behind by increasing equitable access to new and existing vaccines, a vision that China also shares. The vaccine administration law of China was enacted in 2019, which explicitly outlined the establishment of a dynamic mechanism by the National Health Commission and Ministry of Finance to adjust National Immunization Program vaccines in China.^18^ Subsequently, discussions about the incorporation of non-National Immunization Program vaccines in China were postponed due to the outbreak of COVID-19. The global COVID-19 pandemic drew attention to the threats posed by infectious diseases, increasing focus on non-programme vaccines in China's public health agenda. In 2022, the State Council of China announced the 14th Five-Year National Health Plan (2021–25), which included an objective to adjust the composition of programme vaccines in accordance with the country's evolving health-care needs.^19^ Concurrently, the Chinese Center for Disease Control and Prevention (CDC) conducted an analysis of expert opinions on decision-making procedures to introduce non-programme vaccines to China, in which an indicator system of evaluation was constructed.^20^

Financing immunisation programmes used to be a key challenge in China because non-National Immunization Program vaccines were often more expensive than those included in the programme. The budget allocated to procure programme vaccines had been set on the basis of the largest number of newborns predicted to be born in China; however, the population of newborns decreased substantially from 18·83 million in 2016 to 9·56 million in 2022.^11^, ^12^ Therefore, costs of National Immunization Program vaccines decreased accordingly, leading to a surplus of unused funds. Thus, these funds could be further redistributed to support the introduction of non-programme vaccines.

According to the WHO vaccine position papers, PCV, rotavirus vaccine, Hib vaccine, and varicella vaccine (which are categorised as non-National Immunization Program vaccines in China) have been shown to be highly safe and effective against related diseases. WHO recommends that all member countries and regions include PCV, rotavirus vaccine, and Hib vaccine in their immunisation programmes, and countries with a high burden of varicella disease include varicella vaccine.^7^, ^8^, ^9^, ^10^ As of 2021, among the 194 WHO member countries and regions, 162 included PCV in their routine childhood immunisation programmes, 110 included rotavirus vaccine, 193 included Hib vaccine, and 51 included varicella vaccine.^7^, ^8^, ^9^, ^10^ Notably, in 2023, China is the only country among 194 WHO member countries and regions that has not included the Hib vaccine.

In this review, childhood vaccines refer to routine vaccines (included and not included in the National Immunization Program) that are administered in children aged 0–6 years in China. We excluded the non-programme influenza vaccine from our analysis, since influenza vaccine is recommended for a broader range of individuals and is not limited to children younger than 6 years.^21^ Additionally, reliable estimates of long-term disease burden and vaccine cost-effectiveness are scarce for influenza in children in China, which makes it hard to compare influenza vaccine with other vaccines.^22^

WHO has issued principles and considerations, including a range of factors that should be considered for adding a vaccine to immunisation programmes.^23^ These factors encompass an assessment of the prevalence and burden of preventable diseases, the nature of the vaccine, and the strength of the immunisation programme and health-care system. Additionally, these guidelines address essential components of planning and executing the introduction of a vaccine, as well as systematic monitoring and evaluating its effect after implementation. This study focuses on the first step of deciding on vaccine introductions in China. Following WHO's recommendations, we aim to examine four childhood non-National Immunization Program vaccines—namely, PCV13, rotavirus vaccine, Hib vaccine, and varicella vaccine—in terms of coverage rates and their inequalities, disease burden, and cost-effectiveness in China, and to recommend a priority order of non-programme vaccines introduced to the National Immunization Program with the highest benefit to the Chinese population.

## Vaccine coverage and inequalities

A recent study analysed cross-sectional data from ten provinces in China, including vaccine records of 5294 children in 2019, and revealed inadequate coverage rates for the four childhood non-National Immunization Program vaccines (table 1).^6^ Although the absolute number of non-programme vaccines purchased has risen, coverage rates have remained low considering China's large population. For instance, for PCV13, the one-dose coverage rate was 7·7% (95% CI 6·9–8·4) and the three-dose coverage rate was 5·1% (4·5–5·8). The one-dose coverage rate for varicella vaccine was 67·1% (65·4–68·8), the highest among the four non-programme vaccines, partly because a few provinces and cities had included varicella vaccines in local immunisation programmes. According to the vaccine administration law of China, provincial and city governments can include non-programme vaccines in local immunisation programmes.^18^ For instance, since the burden of varicella disease has been particularly high in provinces and cities with large populations in China,^24^ places such as Shanghai and Tianjin included the varicella vaccine in local immunisation programmes. However, such inclusion might have increased regional inequalities since vaccines were free for individuals in regions that subsidised varicella vaccines in local immunisation programmes.

**Table 1:** Market prices, individual costs, coverage rates, and inequalities for childhood non-National Immunization Program vaccines in China, 2019

	**Market price per dose ($)**^22^, ^23^, ^24^, ^25^	Total individual cost per child ($)*	**Vaccine coverage rate (95% CI)**^6^		**Vaccine coverage inequality (95% CI)**^6^	
			One dose	Three doses	One dose	Three doses
PCV13	101·16	314·34	7·7% (6·9–8·4)	5·1% (4·5–5·8)	0·60 (0·57–0·64)	0·64 (0·60–0·68)
Rotavirus vaccine†	26·50	90·36	20·3% (19·2–21·3)	1·8% (1·3–2·2)	0·38 (0·36–0·41)	0·77 (0·71–0·83)
Hib vaccine	11·62	45·72	42·6% (41·3–44·0)	25·0% (23·7–26·3)	0·29 (0·28–0·31)	0·43 (0·41–0·45)
Varicella vaccine‡	20·79	24·41	67·1% (65·4–68·8)	..	0·13 (0·12–0·15)	..

PCV13=13-valent pneumococcal conjugate vaccine. Hib=*Haemophilus influenzae* serotype b.

* Individuals were charged an additional cost of CN¥25 ($3·62) per dose for an immunisation service fee for non-National Immunization Program vaccines.

† The predominant rotavirus vaccine in 2019 was the lamb rotavirus vaccine, so its corresponding market price per dose for that year was used.

‡ One dose of varicella vaccine was often administered in China.

For complete immunisation, based on the market prices of non-National Immunization Program vaccines in China in 2019,^25^, ^26^, ^27^, ^28^ the total personal costs per child in China's private market was $314·34 for three doses of PCV13, $90·36 for three doses of rotavirus vaccine, $45·72 for three doses of Hib vaccine, and $24·41 for one dose of varicella vaccine. There was a negative correlation between the coverage rates of the four non-programme vaccines and their prices per dose or total individual costs per child. Coverage was also strongly associated with household income (table 1).^6^ The concentration index can range from –1 to 1**,** with higher values indicating greater coverage inequalities. The positive concentration index showed that people with a high income received more non-programme vaccines than people with a low income. For three doses of PCV13 and rotavirus vaccine, coverages were low, and inequalities were high, which were reflected by high concentration indices (0·64 [95% CI 0·60–0·68] for PCV13 and 0·77 [0·71–0·83] for rotavirus). The concentration index for three doses of Hib vaccine was 0·43 (95% CI 0·41–0·45). Non-programme varicella vaccine had the lowest one-dose concentration index of 0·13 (0·12–0·15). By contrast, concentration indices were small for three doses of DTP vaccine (0·016 [0·012–0·020]), three doses of Polio vaccine (0·011 [0·008–0·014]), and one dose of MMR vaccine (0·007 [0·005–0·010]), suggesting minimal inequalities in their coverage as National Immunization Program vaccines.

Low coverage rates of and high inequalities in childhood non-National Immunization Program vaccines might be attributed to several factors in China. First, non-programme vaccines were often newly developed and expensive, which restricted access to vaccines, particularly among populations and regions with fewer financial resources.^6^, ^29^ Second, poor awareness and vaccine hesitancy might have contributed to low vaccination rates as many non-programme vaccines were new and might not have been adequately addressed in vaccination campaigns.^6^ Lastly, the supply of non-programme vaccines were slow to respond to changes in demand due to technological challenges and a shortage of market incentives, especially in poor areas.^1^

We found that inequalities in coverage of the four childhood non-National Immunization Program vaccines in China were positively correlated with their prices per dose and total individual costs of complete immunisation per child. Low-income provinces generally had lower coverage rates of non-programme vaccines and higher levels of socioeconomic inequalities.^6^ If these non-programme vaccines were included in the National Immunization Program at the national level, inequalities in coverage would be substantially reduced in China.

## High burden of vaccine-preventable diseases

Statistics have indicated that pneumonia and diarrhoea remain the only potentially vaccine-preventable diseases among the top ten causes of deaths in children younger than 5 years in China: other causes of mortality are congenital diseases and accidents.^30^ A considerable proportion of deaths from childhood pneumonia could be attributed to *Streptococcus pneumoniae* and Hib disease; however, the use of PCV and Hib vaccines show promising results in preventing such fatalities.^31^, ^32^ Rotavirus vaccine has also proven effective in preventing deaths related to rotavirus diarrhea.^33^ According to the 2019 National Notifiable Infectious Disease Surveillance System, varicella was the third most frequently reported vaccine-preventable infectious disease in China, with 981 700 cases documented after tuberculosis and influenza.^28^

Researchers have developed statistical models specifically for China to estimate the burden of these childhood diseases of pneumococcus (preventable by PCV13), Hib disease, rotavirus diarrhoea, and varicella disease while considering vaccine effectiveness and serotype coverage for different pathogens.^25^, ^26^, ^27^, ^28^, ^31^ Using evidence from Global Burden of Disease (GBD) all-cause data, vaccine clinical trials, surveillance studies of bacterial meningitis, and pathogen-specific case fatality ratios, Zhang and colleagues^25^ and Lai and colleagues^31^ estimated pneumonia, meningitis, and non-pneumonia, non-meningitis preventable deaths and cases by PCV13 and Hib vaccine in children younger than 5 years in China in 2017. Lai and colleagues updated the estimated disease burden for 2019.^26^ In another study, Wang and colleagues^27^ estimated the number of preventable rotavirus deaths and cases of gastroenteritis in children younger than 5 years with various rotavirus vaccines in China on the basis of 2019 GBD data, the UN Interagency Group for Child Mortality Estimation, the China National Maternal and Child Health Surveillance System, systematic reviews, and population-based surveillance data. Feng and colleagues used a dynamic transmission model to estimate disease burden of varicella disease and the effect of varicella vaccination in China in 2019.^28^ The burdens of pneumococcal disease, rotavirus diarrhoea, and Hib disease were estimated on the basis of a cohort of newborn children aged 1–59 months, as these diseases primarily affect children younger than 5 years.^25^, ^26^, ^27^, ^31^ The burden of varicella disease was estimated for an annual average of the entire population between 2019 and 2049, as varicella disease could occur in any age group, although it predominantly affects children.^28^ These studies modelled disease burden associated with childhood non-National Immunization Program vaccines using a status quo scenario (ie, a scenario in which these vaccines are used in the private market as non-programme vaccines) and a scenario after vaccine inclusion to China's National Immunization Program.^25^, ^26^, ^27^, ^31^

We focused on the burden of deaths extracted from previous literature, which provided similar data across different pathogens. In 2019, in children younger than 5 years, 114 270 deaths occurred including neonatal deaths and 62 995 deaths occurred excluding neonatal deaths.^34^ Of all 114 270 deaths, 7234 deaths (95% CI 4919–8307) were associated with pneumococcal disease,^26^ 5855 deaths (4476–7530) were associated with rotavirus gastroenteritis,^27^ and 2915 deaths (2737–3094) were associated with Hib disease,^25^, ^31^ accounting for 14·01% of deaths in children younger than 5 years including newborn deaths, or 25·41% of deaths in children younger than 5 years excluding newborn deaths. Pneumococcal disease (preventable by PCV13) had the highest estimated burden of deaths in 2019 (table 2).^26^ No associated deaths were estimated for varicella disease in China.^35^, ^36^

**Table 2:** Disease burden preventable by four childhood non-National Immunization Program vaccines in China

	**Disease and vaccine**	Disease burden with status quo scenario (95% CI)*		**Disease burden with the National Immunization Program scenario (95% CI)**†		**Difference (95% CI)**		**Reduction in deaths in children younger than 5 years in 2019 (%)**	
		**Inpatient cases (n=617 199)**	**Deaths (n=16 004)**	**Inpatient cases (n=171 478)**	**Deaths (n=4243)**	**Inpatient cases (n=445 721)**	**Deaths (n=11 761)**	**Including newborn deaths**	**Excluding newborn deaths**
Lai et al (2022)^26^	Pneumococcal disease (PCV13)‡	185 879 (154 784–224 713)	7234 (4919–8307)	62 527 (52 071–75 587)	2427 (1651–2787)	123 352 (102 712–149 126)	4807 (3269–5520)	4·21%	7·63%
Wang et al (2022)^27^	Gastroenteritis (pentavalent rotavirus vaccine)§	366 661 (280 301–471 533)	5855 (4476–7530)	100 453 (76 793–129 184)	1604 (1226–2063)	266 208 (203 508–342 349)	4251 (3250–5467)	3·72%	6·75%
Zhang et al (2021);^25^ Lai et al (2022)^31^	Hib disease (Hib vaccine)¶	50 682 (48 630–52 732)	2915 (2737–3094)	3963 (3803–4123)	212 (199–225)	46 719 (44 827–48 609)	2703 (2538–2869)	2·37%	4·29%
Feng et al (2023)^28^	Varicella disease (varicella vaccine)‖	13 977 (13 327–14 627)	0	4535 (2626–6443)	0	9442 (9003–9881)	0	0	0

PCV13=13-valent pneumococcal conjugate vaccine. Hib=*Haemophilus influenzae* serotype b. NPNM=non-pneumonia non-meningitis.

* Status quo strategies (ie, non-National Immunization Program vaccines voluntarily used by citizens in the private market but not provided by governments as National Immunization Program vaccines) referred to three doses of PCV13, three doses of Hib vaccine, three doses of lamb rotavirus vaccine, and one dose of varicella vaccine.

† Programme strategies referred to three doses of PCV13, three doses of Hib vaccine, three doses of pentavalent rotavirus vaccines, and one dose plus catch-up of varicella vaccine.

‡ Inpatients with pneumococcal disease included inpatient pneumonia, meningitis, and NPNM due to streptococcus pneumoniae. Pneumococcal deaths included deaths of inpatient pneumonia, meningitis, and NPNM due to streptococcus pneumoniae. All PCV13 products available in the market were assumed to have the same effectiveness.

§ Gastroenteritis deaths included deaths of inpatient gastroenteritis attributed to rotavirus.

¶ Hib inpatient cases included inpatient pneumonia, meningitis, and NPNM due to Hib. Hib deaths included deaths of inpatient pneumonia, meningitis, and NPNM due to Hib. All Hib vaccine products available in the market were assumed to have the same effectiveness. Hib disease burden estimates were derived from data from 2017.

‖ No varicella deaths were assumed. All varicella vaccine products available in the market were assumed to have the same effectiveness.

Previous studies showed that the inclusion of PCV13, rotavirus vaccine, and Hib vaccine in China's National Immunization Program in 2019 could have prevented 11 761 deaths among children younger than 5 years (table 2),^25^, ^26^, ^27^, ^31^ which accounted for 10·29% of total national deaths in this age group.^34^ If newborn deaths not preventable by vaccines were excluded, the percentage would increase to 18·67%. The inclusion of these three non-programme vaccines would have reduced mortality in children younger than 5 years from 7·8 per 1000 to 7·0 per 1000, surpassing the actual total mortality reduction observed in 2 years from 7·8% in 2019 to 7·1% in 2021 (see calculation in the appendix, pp 3–5).^34^ Additionally, these studies showed that the four non-programme vaccines would have reduced inpatient admissions by 455 721 patients in 2019 (table 2).^25^, ^26^, ^27^, ^28^, ^31^

## Cost-effectiveness

Previous studies have used health economic methods and compared the incremental cost-effectiveness ratio of each childhood non-National Immunization Program vaccine with the status quo scenario.^25^, ^26^, ^27^, ^28^ Costs included expenses on vaccine procurement and immunisation services. Quality-adjusted life-years (QALYs) were used as effectiveness measures. However, none of the existing studies directly compared all four non-programme vaccines together to rank their respective cost-effectiveness, even if they used similar methods. The assessments were conducted from a societal perspective, assuming that vaccine prices in the status quo scenario would remain unchanged in the National Immunization Program. Since incidence rates of PCV and non-vaccine serotypes fluctuated due to herd effects and serotype replacement as vaccine coverage increased,^37^ Lai and colleagues^31^ modelled PCV13 costs and effects within 5 years after its introduction and used pre-PCV13 and post-PCV13 introduction incidence rate ratios (IRRs) instead of vaccine efficacy, which was done in the status quo scenario.

In accordance with criteria for vaccine cost-effectiveness analyses outlined by WHO, a vaccine would be considered highly cost-effective if the incremental cost-effectiveness ratio was less than the country's gross domestic product (GDP)/capita and cost-effective if the incremental cost-effectiveness ratio was between one and three times the GDP/capita.^23^ The cost-effective thresholds based on health opportunity costs were $3650–5669 in China,^38^ which is consistent with findings in another study.^39^ Additionally, the threshold based on the value of statistical life in China was estimated to be 1·45 times the GDP/capita.^40^ The WHO threshold has been applied in previous literature in China.^23^ In the present study other thresholds were used to check the robustness of the cost-effectiveness results.

If the following vaccines had been included in the National Immunization Program in 2019, the averted QALYs lost in children younger than 5 years would have been 146 366 for PCV13, 131 850 for pentavalent rotavirus vaccine, 85 388 for Hib vaccine, and 6944 for varicella vaccine (table 3).^25^, ^26^, ^27^, ^28^ The incremental cost-effectiveness ratio of PCV13 at market price was $18 628/QALY, exceeding China's GDP/capita ($10 276) in 2019. The incremental cost-effectiveness ratio was $8836/QALY for pentavalent rotavirus vaccine and $7999/QALY for Hib vaccine. Interestingly, the incremental cost-effectiveness ratio for the varicella vaccine in 2019 was negative, indicating cost-saving results. These previous economic analyses using market vaccine prices showed that PCV13 was cost-effective, whereas pentavalent rotavirus and Hib vaccines were substantially cost-effective based on the thresholds of WHO and the value of statistical life in China.

**Table 3:** Cost-effectiveness of childhood non-National Immunization Program vaccines in China

	**Vaccine**	**No addition of non-programme vaccines (status quo scenario)**		**Addition of non-programme vaccines**		**Difference**		ICER at market price ($/QALY)*	**ICER at 50% reduced market price ($/QALY)**†
		Costs ($ million)‡	Loss of QALYs§	Costs ($ million)‡	Loss of QALYs§	Costs ($ million)‡	QALYs§		
Lai et al (2023)^26^	PCV13	1389	241 602	4115	95 266	2726	146 336	18 628	8037
Wang et al (2022)^27^	Rotavirus (pentavalent) vaccine	1660	183 435	2825	51 585	1165	131 850	8836	1550
Zhang et al (2021)^25^	Hib vaccine	760	92 231	1443	6843	683	85 388	7999	4447
Feng et al (2023)^28^	Varicella vaccine	569	10 436	552	3492	−17	6944	−2448	−18 916

ICER=incremental cost-effectiveness ratio. QALY=quality-adjusted life years. PCV13=13-valent pneumococcal conjugate vaccine. Hib=*Haemophilus influenzae* serotype b.

* The market prices of non-National Immunization Program vaccines in 2019 were assumed to remain the same in the programme.

† The market prices of non-National Immunization Program vaccines in 2019 were assumed to reduce by 50% after their inclusion in the programme. No changes were made to any other costs.

‡ Costs for PCV13, rotavirus vaccine, and Hib vaccine included costs of disease treatment, indirect costs of diseases, and costs of immunisation for the 2019 cohort of newborn children. Costs for varicella vaccine included annual mean costs of disease treatment, indirect costs of diseases, and costs of immunisation in 2019–49.

§ QALYs for PCV13, Hib vaccine, and rotavirus vaccine were for all newborns in the 2019 cohort in their first 5 years of life. QALYs for varicella vaccine were annual mean QALY loss due to varicella disease for the entire population in 2019–49, as dynamic epidemiological modelling considered the possibility of varicella disease affecting individuals outside the vaccinated cohort.

Since 2018, the national centralised drug procurement has reduced medicine prices by more than 50% in China.^41^ A similar national, centralised approach for vaccine procurement has been implemented for National Immunization Program vaccines since 2020, leading to lower programme vaccine prices. The central government in China funds National Immunization Program vaccination in full, ensuring widespread access. Including childhood non-programme vaccines in the National Immunization Program is likely to reduce costs of these vaccines through government bidding and procurement. For example, assuming a 50% reduction in procurement prices, the incremental cost-effectiveness ratio of PCV13 would decrease to $8037/QALY, which would be considered highly cost-effective on the basis of the WHO Commission on Macroeconomics and Health thresholds.^23^, ^40^, ^42^ Additionally, the incremental cost-effectiveness ratio would be $1550/QALY for pentavalent rotavirus vaccine and $4447/QALY for Hib vaccine, indicating high cost-effectiveness. Based on these cost-effectiveness results with an assumption of a 50% reduction in market prices, we suggest a recommended priority order for the four childhood non-programme vaccines of varicella vaccine, pentavalent rotavirus vaccine, Hib vaccine, and PCV. Notably, in the cost-effectiveness study of PCV13 by Lai and colleagues,^26^ a three-dose schedule was modelled following the recommendation of the latest WHO position paper.^7^ Other vaccination schedules might be considered, such as a 1 plus 1 schedule as observed in the UK. However, empirical evidence is insufficient in China to quantify the efficacy of these alternative vaccination schedules. It would be challenging to predict its feasibility and cost-effectiveness, although it is intuitively apparent to save vaccination costs with vaccination schedules with fewer doses.

## Multicriteria decision analysis to optimise priority ranking

We investigated the overall priority order of four non-National Immunization Program vaccines using multicriteria decision analysis (MCDA), considering four dimensions of disease burden, vaccine coverage, inequalities, and cost-effectiveness.^43^, ^44^, ^45^, ^46^, ^47^ In 2022, the Chinese CDC analysed the weights for introducing non-programme vaccines in China with experts from the fields of vaccine, epidemiology, infectious diseases, health economics, and related disciplines. Based on this analysis, the overall weight was 40% for disease burden, 20% for vaccine coverage, 20% for inequalities, and 20% for cost-effectiveness.^20^ We used these weights to conduct an MCDA and optimise the priority order of the four non-programme vaccines in China (table 4). Using the current market prices of these vaccines (table 1), we calculated the MCDA scores for PCV13 to be highest at 1·066, followed by rotavirus vaccine (0·649), Hib vaccine (0·353), and varicella vaccine (0·233; method provided in the appendix, pp 6–9). If the prices of non-programme vaccines were reduced by 50% after their inclusion in the National Immunization Program, the priority order would remain the same, with a higher MCDA score for PCV13 (1·244), but a lower MCDA score for varicella vaccine (0·089). The MCDA scores for rotavirus and Hib vaccines showed minimal variations in response to the reduction in price.

**Table 4:** MCDA of priority ranking for including four childhood non-National Immunization Program vaccines in China's National Immunization Program

	**At current market price**	**At 50% reduced market price**
PCV13	1·066	1·244
Rotavirus vaccine	0·649	0·644
Hib vaccine	0·353	0·339
Varicella vaccine	0·233	0·089

Calculation provided in the appendix (pp 6–9). MCDA=multicriteria decision analysis. PCV13=13-valent pneumococcal conjugate vaccine. Hib=*Haemophilus influenzae* serotype b.

## Effect on budget

Considering the high prices of non-programme vaccines in China, it is crucial to assess the financial implications of adding any vaccines to the National Immunization Program. We assumed that the newborn cohort in 2023 will resemble that of 2022 when we estimated the effect on government budget of including childhood non-programme vaccines in the National Immunization Program. In alignment with WHO's recommendations, we followed a three-dose vaccination strategy for PCV13, rotavirus vaccine, and Hib vaccine in the National Immunization Program.^7^, ^8^, ^9^, ^10^ For varicella vaccination, we used a one-dose plus mass catch-up strategy as it was the most cost-effective method in China;^28^ the two-dose strategy had higher government costs. The total government costs included both vaccine procurement costs borne by the central government and immunisation service costs covered by local governments (ie, human resources, transportation, cold chains, surveillance, communication, training, and supervision at various government levels), which were estimated marginal costs after introducing non-programme vaccines to the National Immunization Program.^48^

The market prices of non-programme vaccines in 2022 were used to estimate the government costs in 2023.^49^ We estimated that total government costs for newborns in 2023 were $1954·92 million for PCV13, $1273·13 million for pentavalent rotavirus vaccine, $415·30 million for Hib vaccine, and $221·64 million for varicella vaccine (table 5; appendix pp 10–11). An additional one-time expense of $1368·18 million would be required for catch-up vaccinations in children aged 1–12 years who were still susceptible to varicella diseases. Of these government costs, approximately 94·95% would be allocated to PCV13 procurement, 92·25% to pentavalent rotavirus vaccine procurement, 76·25% to Hib vaccine procurement, and 85·17% to varicella vaccine procurement.

**Table 5:** Budget impact from the government perspective of introducing four childhood non-National Immunization Program vaccines in China's National Immunization Program

		**Children (million)**	Total doses (million)*	**Market price per dose in 2022 ($)**	**Total vaccine purchase costs for the central government ($ million)**†	**Immunisation service costs for the local government ($ million)**‡	**Total programme costs at market price ($ million)**	**Total programme costs at 50% reduced market price ($ million)**
PCV13		9·56	27·25	68·12	1856·27	98·65	1954·92	1026·79
Pentavalent rotavirus vaccine		9·56	27·25	43·10	1174·48	98·65	1273·13	685·89
Hib vaccine		9·56	27·25	11·62	316·65	98·65	415·30	256·98
Varicella vaccine								
	Newborn vaccine	9·56	9·08	20·79	188·77	32·87	221·64	127·26
	Catch-up vaccine	59·00	56·05	20·79	1165·28	202·90	1368·18	785·54

PCV13=13-valent pneumococcal conjugate vaccine. Hib=*Haemophilus influenzae* serotype b.

* We assumed a vaccination rate of 95% for all vaccines in China's National Immunization Program.

† The slight inconsistency in results might be attributed to rounding errors.

‡ An additional $3·62 (CN¥25)/dose was charged as the immunisation service fee.

Prices of non-programme vaccines tend to be higher than those of vaccines included in China's National Immunization Program because they are purchased outside the national centralised system for vaccine procurement.^15^ Although the same vaccines can be procured as programme or non-programme vaccines in China, their prices differ greatly. For instance, in 2022, the government paid $3·96/dose for inactivated HAV vaccine (0·5 mL/250 IU), $1·17/dose for HBV vaccine (0·5 mL/10 μg), and $4·46/dose for IPV vaccine (0·5 mL) as programme vaccines.^15^ By contrast, the lowest out-of-pocket prices for citizens in the private market in 2022 were $20·82/dose for inactivated HAV vaccine, $13·28/dose for HBV vaccine, and $26·46/dose for IPV, as non-programme vaccines in China.^49^ After incorporating non-programme vaccines into the National Immunization Program, it is anticipated that National Centralized Vaccine Procurement will be used to procure all programme vaccines.^49^ This process involves selecting a few manufacturers for each programme vaccine, and ensuring that their production capacity will cater to the national population's vaccine demands. Additionally, the dose quantity and price of programme vaccines for the country is calculated. The substantial volume of programme vaccines procured leads to economies of scale for manufacturers and reduces uncertainty. By contrast, the current provincial procurement for non-programme vaccines only sets prices, whereas specific dose quantities for each manufacturer are not specified. Instead, dose quantities are often limited and determined by contracts between county or district branches of the Chinese CDC and manufacturers. Furthermore, many manufacturers are involved in the procurement process of non-programme vaccines, and they generally do not have a clear expectation of the required dose quantities.^49^ If the procurement prices of non-programme vaccines decrease after being added to the National Immunization Program, the government costs for vaccine procurement will substantially decrease, resulting in improved incremental cost-effectiveness ratios. Moreover, new vaccines added to the National Immunization Program will share the same immunisation delivery system in local areas. This, in turn, might reduce immunisation delivery costs for local governments through economies of scale and improved efficiency of capacity use in the immunisation system.

The costs of introducing new vaccines to the National Immunization Program could be offset by savings from the procurement of National Immunization Program vaccines. These savings could be due to a smaller birth cohort, lower vaccine prices achieved through the national centralised vaccine procurement, and additional funding from the Ministry of Finance. The four non-programme childhood vaccines could be included in the National Immunization Program one at a time to avoid sudden large budget increases. Vaccine procurement and service costs would jointly be covered by the central government and local governments according to the financing policy of China's National Immunization Program. It will be important to establish surveillance mechanisms to monitor disease burden (including the effect on health inequalities), vaccine coverage, adverse events following immunisation, and the actual vaccine delivery costs for the new vaccines. Since non-programme vaccines have already been used in the private market for several years in China, minimal additional training for health-care workers would be required.

This study has several limitations that warrant discussion. First, we conducted a comprehensive review of existing literature pertaining to non-National Immunization Program vaccines in China, specifically PCV13, rotavirus vaccine, Hib vaccine, and varicella vaccine, focusing on factors such as preventable disease burdens, vaccine coverage, inequalities, and cost-effectiveness. However, these individual studies might use varied methods, data inputs, and analytical approaches, which poses challenges when attempting to draw entirely comparable conclusions. Second, previous literature estimating the potential reduction in mortality in children younger than 5 years driven by the introduction of each non-programme vaccine in China might be overestimated. This could be attributed to the possibility of double counting, as children who did not receive all these non-programme vaccines were not susceptible to multiple pathogens simultaneously. Lastly, it would be worthwhile to conduct further estimations at the subnational level for the introduction of non-programme vaccines, as the enactment of China's vaccine administration law in 2019 granted Chinese provinces and cities the autonomy to include new vaccines. We acknowledged the potential for subnational analyses as a promising avenue for future research.

## Conclusion

In this review of China's non-National Immunization Program vaccines (PCV13, rotavirus vaccine, Hib vaccine, and varicella vaccine) that assesses disease burden, coverage, inequalities, cost-effectiveness, multicriteria decision analysis, and calculation of governmental budgets, we concluded a priority order for the four childhood vaccines to be introduced to China's National Immunization Program. If the primary goal of including these vaccines in the programme is to improve vaccination coverage rates and decrease inequalities, they should be introduced in the following priority order: PCV13, rotavirus vaccine, Hib vaccine, and varicella vaccine. The considerable burden of vaccine-preventable diseases among children in China is closely correlated with low and unequal coverage of non-programme vaccines, where pneumococcal disease imposes the highest disease burden. The multicriteria decision analysis considering disease burden, coverage rates, inequalities, and cost-effectiveness suggested the same optimal sequence for introducing childhood non-programme vaccines in China.

China has made substantial progress in vaccination, achieving consistently high coverage rates for existing vaccines in the National Immunization Program.^1^ The number of National Immunization Program vaccine doses administered has been reducing, mainly due to a decline in the number of newborns.^11^, ^12^, ^34^ The total procurement costs for programme vaccines remain relatively small compared with the overall national health expenditures in China.^15^, ^34^ The disease burdens preventable by the non-programme vaccines in China lacked comprehensive documentation in previous literature, so the health authorities did not possess sufficient evidence to justify their inclusion in the National Immunization Program. However, several recent studies have shed light on the substantial disease burden in China.^25^, ^26^, ^27^, ^28^, ^31^ Although PCV13, rotavirus vaccine, and Hib vaccine have been recommended by WHO,^7^, ^8^, ^9^ they are not included in China's National Immunization Program and require 100% personal payments for access. Consequently, the coverage for these vaccines remains low and unequal, posing challenges to their widespread use.^6^ These vaccines could substantially reduce disease burden and decrease the mortality rate among children younger than 5 years. To address the issue of affordability and accessibility, it is crucial to explore the possibility of reducing the prices of childhood non-programme vaccines in China through national central vaccine procurement.^49^ Moreover, to ensure equitable immunisation coverage and further enhance the National Immunization Program in China, we recommend adding all four childhood non-programme vaccines to the National Immunization Program using a step-by-step strategy. Every eligible child would have access to these vaccines for free, leaving no child behind in the pursuit of better health. Adding childhood non-programme vaccines to China's National Immunization Program would also constitute a substantial contribution to achieving the goals of Healthy China 2030 and Immunisation Agenda 2030.^17^, ^19^

### Search strategy and selection criteria

We searched PubMed and Google Scholar for terms including “vaccine”, “immunization”, “category 1 vaccines”, “NIP vaccines”, “category 2 vaccines”, “non-NIP vaccines”, “routine immunization”, “national immunization program (NIP)”, “immunization program”, “vaccine coverage”, “vaccination coverage”, “vaccine-preventable diseases”, “disease burden”, “vaccine equity”, “cost-effectiveness”, “health policy”, “health economic”, “vaccine financing”, “vaccine procurement”, “immunization challenges”, and “China” for articles published in English between Jan 1, 2010, and May 1, 2023. We also searched the China National Knowledge Infrastructure using the same date restrictions and terms in Chinese for Chinese articles. Identified publications were screened for relevance on the basis of their titles and abstracts. Additional relevant articles were obtained from references of selected publications and websites of major organisations, including WHO and the Chinese Government. 63 English articles and 32 Chinese articles were considered in the final review. All literature was evaluated and underwent a synthesis process to formulate health policy recommendations on the introduction of non-National Immunization Program vaccines in China.

For **market prices of vaccines** see https://www.yaozh.com/

## Declaration of interests

HF received funding from the Bill & Melinda Gates Foundation and Sanofi. MJ received funding from the National Institute for Health and Care Research, RCUK, the Bill & Melinda Gates Foundation, GAVI, the European Union, and the Wellcome Trust. BNP received funding from GAVI, WHO, United States Agency for International Development, NSF, Vaxart, Copenhagen Consensus Center, and Costello Medical. HZ received funding from WHO. XL declares no competing interests.

## References

[1] Chen S, Yao L, Wang W, Tang S (2022). Developing an effective and sustainable national immunisation programme in China: issues and challenges. Lancet Public Health.

[2] (1985). World Health Assembly (27th: 1974). The expanded programme on immunization: the 1974 resolution by the World Health Assembly. Assignment Child.

[3] Yu W, Lee LA, Liu Y (2018). Vaccine-preventable disease control in the People's Republic of China: 1949–2016. Vaccine.

[4] Liu J, Liang W, Jing W, Liu M (2019). Countdown to 2030: eliminating hepatitis B disease, China. Bull World Health Organ.

[5] WHO (2014). Frequently asked questions on Hepatitis B vaccination in China. https://www.who.int/hongkongchina/news/detail/18-08-2014-frequently-asked-questions-on-hepatitis-b-vaccination-in-china.

[6] Zhang H, Lai X, Mak J (2022). Coverage and equity of childhood vaccines in China. JAMA Netw Open.

[7] WHO (2019). WHO position paper: pneumococcal conjugate vaccines in infants and children under 5 years of age. https://www.who.int/publications/i/item/10665-310968.

[8] WHO (2021). Rotavirus vaccines: WHO position paper. https://www.who.int/publications/i/item/WHO-WER9628.

[9] WHO (2013). Haemophilus influenzae type B (Hib) vaccination position paper. https://www.who.int/publications/i/item/who-wer8839-413-426.

[10] WHO (2014). Varicella and herpes zoster vaccines: WHO position paper. https://www.who.int/publications/i/item/who-wer-8925-265-288.

[11] National Bureau of Statistics of the People's Republic of China (2022). The statistical report of national economy and social development of the People's Republic of China 2022. https://www.gov.cn/xinwen/2023-02/28/content_5743623.htm.

[12] National Bureau of Statistics of the People's Republic of China (2023).

[13] National Institutes for Food and Drug Control of the People's Republic of China (2023). The lot release data of biological products in China. https://www.nifdc.org.cn/nifdc/xxgk/ggtzh/jwgk/shwzhppfgg/index.html.

[14] Lai X, Ma Y, Fang H (2023). Better adherence to childhood Haemophilus influenzae type B vaccination with combination vaccines compared to single-antigen vaccines: evidence from China. J Glob Health.

[15] The State Council of the People's Republic of China (2022). Central government procurement 2022. http://zycg.cn/freecms/site/zygjjgzfcgzx/cggg/index.html.

[16] The State Council of the People's Republic of China (Oct 12, 2023). The 2022 Statistical Bulletin on Health Care Development is released. https://www.gov.cn/lianbo/bumen/202310/content_6908686.htm.

[17] World Health Assembly (2020). Immunization agenda 2030: a global strategy to leave no one behind. https://www.who.int/teams/immunization-vaccines-and-biologicals/strategies/ia2030.

[18] National People's Congress of the People's Republic of China (2019). Vaccine administration law of the People's Republic of China. http://www.npc.gov.cn/npc/c30834/201907/11447c85e05840b9b12c62b5b645fe9d.shtml.

[19] State Council of the People's Republic of China (2021). The 14th five-year national health plan. https://www.gov.cn/zhengce/zhengceku/2022-05/20/content_5691424.htm.

[20] Ma C, Li J, Wang N (2022). Prioritization of vaccines for inclusion into China's expanded program on immunization: evidence from experts' knowledge and opinions. Vaccines (Basel).

[21] WHO (2018). Influenza (seasonal) fact sheet. http://www.who.int/mediacentre/factsheets/fs211/en/.

[22] Hodgson D, Baguelin M, van Leeuwen E (2017). Effect of mass paediatric influenza vaccination on existing influenza vaccination programmes in England and Wales: a modelling and cost-effectiveness analysis. Lancet Public Health.

[23] WHO (2014). Principles and considerations for adding a vaccine to a national immunization programme: from decision to implementation and monitoring. https://www.who.int/publications/i/item/9789241506892.

[24] Wang J, Xu Z, Gao Q (2022). Varicella outbreaks in schools and kindergartens in Shanghai, China from 2011 to 2020. PLoS One.

[25] Zhang H, Garcia C, Yu W (2021). National and provincial impact and cost-effectiveness of Haemophilus influenzae type B conjugate vaccine in China: a modeling analysis. BMC Med.

[26] Lai X, Garcia C, Wu D (2022). Estimating national, regional and provincial cost-effectiveness of introducing childhood 13-valent pneumococcal conjugate vaccination in China: a modelling analysis. Lancet Reg Health West Pac.

[27] Wang J, Zhang H, Zhang H, Fang H (2022). Public health impact and cost-effectiveness of rotavirus vaccination in China: comparison between private market provision and national immunization programs. Hum Vaccin Immunother.

[28] Feng H, Zhang H, Ma C, Zhang H, Yin D, Fang H (2022). National and provincial burden of varicella disease and cost-effectiveness of childhood varicella vaccination in China from 2019 to 2049: a modelling analysis. Lancet Reg Health West Pac.

[29] Yu H, Yang W, Varma JK (2012). To save children's lives, China should adopt an initiative to speed introduction of pneumonia vaccines. Health Aff (Millwood).

[30] Liu Z, Liu XR, He CH (2019). [Analysis of mortality and leading causes of death in Chinese children under 5-year-old between 2010 and 2016]. Zhonghua Yu Fang Yi Xue Za Zhi.

[31] Lai X, Wahl B, Yu W (2022). National, regional, and provincial disease burden attributed to *Streptococcus pneumoniae* and *Haemophilus influenzae* type b in children in China: Modelled estimates for 2010–17. Lancet Reg Health West Pac.

[32] Wahl B, O'Brien KL, Greenbaum A (2018). Burden of Streptococcus pneumoniae and Haemophilus influenzae type b disease in children in the era of conjugate vaccines: global, regional, and national estimates for 2000–15. Lancet Glob Health.

[33] Liu N, Xu Z, Li D, Zhang Q, Wang H, Duan ZJ (2014). Update on the disease burden and circulating strains of rotavirus in China: a systematic review and meta-analysis. Vaccine.

[34] National Health Commission of the People's Republic of China (2020).

[35] Xu Y, Liu Y, Zhang X (2021). Epidemiology of varicella and effectiveness of varicella vaccine in Hangzhou, China, 2019. Hum Vaccin Immunother.

[36] US CDC (2023). Epidemiology and prevention of vaccine-preventable diseases-varicella. https://www.cdc.gov/vaccines/pubs/pinkbook/varicella.html.

[37] Deloria Knoll M, Bennett JC, Garcia Quesada M (2021). Global landscape review of serotype-specific invasive pneumococcal disease surveillance among countries using PCV10/13: the pneumococcal serotype replacement and distribution estimation (PSERENADE) project. Microorganisms.

[38] Ochalek J, Lomas J, Claxton K (2018). Estimating health opportunity costs in low-income and middle-income countries: a novel approach and evidence from cross-country data. BMJ Glob Health.

[39] Woods B, Revill P, Sculpher M, Claxton K (2016). Country-level cost-effectiveness thresholds: initial estimates and the need for further research. Value Health.

[40] Cai D, Shi S, Jiang S, Si L, Wu J, Jiang Y (2022). Estimation of the cost-effective threshold of a quality-adjusted life year in China based on the value of statistical life. Eur J Health Econ.

[41] State Council of the People's Republic of China (2022). Centralized procurement bringing down drug prices. http://english.www.gov.cn/statecouncil/ministries/202208/09/content_WS62f1a4cac6d02e533532ef87.html.

[42] WHO Commission on Macroeconomics and Health & World Health Organization (2001). Macroeconomics and health: investing in health for economic development: executive summary/report of the Commission on Macroeconomics and Health. https://apps.who.int/iris/handle/10665/42463.

[43] Barocchi MA, Black S, Rappuoli R (2016). Multicriteria decision analysis and core values for enhancing vaccine-related decision-making. Sci Transl Med.

[44] Eisenhardt KM, Zbaracki MJ (1992). Strategic decision making. Strateg Manage J.

[45] Thokala P, Devlin N, Marsh K (2016). Multiple criteria decision analysis for health care decision making—an introduction: report 1 of the ISPOR MCDA emerging good practices task force. Value Health.

[46] Frazão TDC, Camilo DGG, Cabral ELS, Souza RP (2018). Multicriteria decision analysis (MCDA) in health care: a systematic review of the main characteristics and methodological steps. BMC Med Inform Decis Mak.

[47] Thokala P, Duenas A (2012). Multiple criteria decision analysis for health technology assessment. Value Health.

[48] Yu W, Lu M, Wang H (2018). Routine immunization services costs and financing in China, 2015. Vaccine.

[49] Zhang H, Patenaude B, Ma C, Fang H (2023). Vaccine pricing strategies in China. BMJ Glob Health.

