# Correction to *Lancet Public Health* 2023; 8: e1016–24

**DOI:** 10.1016/S2468-2667(24)00130-0

**Published:** 2024-06-26

*Zhang H, Lai X, Patenaude BN, Jit M, Fang H. Adding new childhood vaccines to China's National Immunization Program: evidence, benefits, and priorities.* Lancet Public Health *2023; **8:** e1016–24*—In this Health Policy, the multicriteria decision analysis (MCDA) scores in table 4 have been corrected. The corresponding section within the manuscript (sentences 5 and 6 of the section entitled Multicriteria decision analysis to optimise priority ranking) is now: “Using the current market prices of these vaccines (table 1), we calculated the MCDA score for PCV13 to be highest at 0·800, followed by rotavirus vaccine (0·667), Hib vaccine (0·390), and varicella vaccine (0·234; method provided in the appendix, pp 6–9). If the prices of non-programme vaccines were reduced by 50% after their inclusion in the National Immunization Program, the priority order would remain the same, with the same MCDA score for PCV13 of 0·800, but with slightly lower scores for the other vaccines.” The final sentence of section entitled Multicriteria decision analysis to optimise priority ranking has been deleted. The appendix has also been corrected. These corrections have been made as of June 26, 2024.

