# Supplementary material for: Adding new childhood vaccines to China's National Immunization Program: evidence, benefits, and priorities
Source: Lancet Public Health. 2023 Nov 22;8(12):e1016–24. doi: 10.1016/S2468-2667(23)00248-7 (PMC10695764; doi:10.1016/S2468-2667(23)00248-7)
Supplement: Supplementary appendix [file mmc1.pdf]

THE LANCET
Public Health

Supplementary appendix

This appendix formed part of the original submission and has been peer reviewed. We post it as supplied by the authors.

Supplement to: Zhang H, Lai X, Patenaude BN, Jit M, Fang H. Adding new childhood vaccines to China's National Immunization Program: evidence, benefits, and priorities. *Lancet Public Health* 2023; **8:** e1016–24.

Webappendix

Adding New Childhood Vaccines to China's National Immunization Program: Evidence, Benefits, and Priorities

Haijun Zhang [1,2,3#], MS; Xiaozhen Lai [1,4#], BA;
Bryan N. Patenaude [2,5], ScD; Prof. Mark Jit [6,7,8], PhD; Prof. Hai Fang [9,10*], PhD

# Contributed equally
* The corresponding author

Affiliations:
1 Department of Health Policy and Management, School of Public Health, Peking University, Beijing, China
2 International Vaccine Access Center, Bloomberg School of Public Health, Johns Hopkins University, Baltimore, USA
3 Department of Immunization, Vaccines and Biologicals, World Health Organization, Geneva, Switzerland
4 Health Economics Research Centre, Nuffield Department of Population Health, University of Oxford, Oxford, UK
5 Department of International Health, Bloomberg School of Public Health, Johns Hopkins University, Baltimore, USA
6 Department of Infectious Disease Epidemiology, Faculty of Epidemiology and Population Health, London School of Hygiene and Tropical Medicine, London, UK.
7 Centre for Mathematical Modelling of Infectious Diseases, London School of Hygiene and Tropical Medicine, London, UK.
8 School of Public Health, University of Hong Kong, Hong Kong SAR, China.
9 China Center for Health Development Studies, Peking University, Beijing, China
10 Peking University Health Science Center - Chinese Center for Disease Control and Prevention Joint Research Center for Vaccine Economics, Peking University, Beijing, China

Correspondence to:
Prof Hai Fang, China Center for Health Development Studies, Peking University, Haidian District, Beijing 100191, China
hfang@hsc.pku.edu.cn

Contents of Webappendix

Webappendix 1: Percentage of under-five children deaths potentially prevented by non-NIP vaccines in China in 2019 3

Webappendix 2: Multi-Criteria Decision Analysis (MCDA) of introducing non-NIP vaccines into China's NIP 6

Webappendix 3: Governmental budget impact analysis of introducing non-NIP vaccines into China's NIP 10

Webappendix 4: The comprehensive list of citations for individual studies from the existing literature 12

Webappendix 1: Percentage of under-five children deaths potentially prevented by non-NIP vaccines in China in 2019

Recent literature has estimated the disease burden among under-five children in China, which could have been mitigated through the use of non-NIP vaccines. These recent studies analyzed two scenarios in China in 2019. One scenario represented the *status quo*, in which non-NIP vaccines were available in the private market, requiring 100% self-payment, and the real-world coverage rates of non-NIP vaccines were employed in the modelling. The other scenario assumed the inclusion of non-NIP vaccines in the NIP with an assumed coverage rate of 95%, matching the average coverage rates of NIP vaccines in China in 2019.

1. These studies[1-3] estimated the deaths of under-five children in China in 2019 in the scenario of *status quo*, and were cited both in the main text and table 2. In 2019, deaths associated with specific pathogens: pneumococcus (preventable by PCV13), rotavirus (preventable by pentavalent rotavirus vaccine), and Hib (preventable by Hib vaccine) were 7,234[1], 5,855[2], and 2,915[3] respectively (table 2 in the main manuscript). It was assumed that no under-five children deaths were associated with varicella in China[4]. The total number of under-five children deaths associated with these pathogens that could have been prevented by three non-NIP vaccines amounted to 16,004 in China in 2019 (table 2 in the main manuscript). The China Health Statistical Yearbook 2020 reported the neonatal (newborn) death rate (3.5‰) and the under-five children death rate (7.8‰), alongside the total number of live births (14.65 million) in China in 2019. We calculated that the total number of neonatal deaths and under-five children deaths in China in 2019 were 51,275 and 114,270, respectively[5]. Further calculations allowed us to determine that the percentage of under-five children deaths associated with these pathogens in the total number of under-five children deaths equaled to 14.01% according to Formula 1.

   Formula 1:
   Percentage of under-five children deaths associated with these pathogens = [Under-five children deaths associated with pathogens] / [total under-five children deaths in China] * 100% = 16,004 / 114,270 * 100% = 14.01%

2. These studies[1-3] also estimated under-five children deaths in China in 2019 in the scenario of introducing these non-NIP vaccines into the NIP. The inclusion of PCV13, rotavirus, and Hib vaccines in China's NIP in 2019 could have prevented a total of 11,761 deaths among under-five children (4,807 by PCV13, 4,251 by pentavalent rotavirus vaccine, and 2,703 by Hib vaccine) reported in table 2. The number of under-five children deaths preventable by non-NIP vaccines was smaller than that of the total under-five children deaths associated with these pathogens, as vaccines were not 100% effective. We calculated that the percentage of under-five children deaths that could have been prevented by these non-NIP vaccines in the total number of under-five children deaths equaled to 10.29% according to Formula 2.

   Formula 2:
   Percentage of under-five children deaths prevented by non-NIP vaccines = [Under-five children

deaths prevented by non-NIP vaccines] / [total under-five children deaths in China] * 100% = 11,761 / 114,270 * 100% = 10.29%

3. Because neonatal deaths (occurring within 28 days of birth) were not able to be prevented by these non-NIP vaccines, as children were not immunized yet, we excluded the number of neonatal deaths from the total number of under-five deaths in China in 2019, which was used as the denominator (114,270 - 51,275 = 62,995) in Formula 3.

   Formula 3:
   Percentage of under-five children deaths prevented by non-NIP vaccines excluding neonatal deaths = [Under-five children deaths prevented by non-NIP vaccines] / [total under-five children deaths excluding neonatal deaths] * 100% = 11,761/ 62,995 * 100% = 18.67%

   We found that the percentage calculated by Formula 3 was higher than that calculated by Formula 2, as neonatal deaths were excluded from the denominator.

4. There were 14.65 million live births and 114,270 under-five children deaths in China in 2019. The under-five children mortality rate in 2019 was 7.8‰. The inclusion of PCV13, rotavirus vaccine, and Hib vaccine could have prevented a total of 11,761 under-five children deaths. We calculated that these three non-NIP vaccines could potentially decrease the under-five children mortality rate in 2019 from 7.8‰ to 7.0‰ with Formula 4.

   Formula 4:
   Under-five children mortality rate if three non-NIP vaccines were included = [114,270 – 11,761] / 14,650,000 * 1000‰ = 7.0‰

References

[1] Lai X, Garcia C, Wu D, et al. Estimating national, regional and provincial cost-effectiveness of introducing childhood 13-valent pneumococcal conjugate vaccination in China: a modelling analysis. *Lancet Reg Health West Pac* 2023; 32: 100666.

[2] Wang J, Zhang H, Zhang H, Fang H. Public health impact and cost-effectiveness of rotavirus vaccination in China: comparison between private market provision and national immunization programs. *Hum Vaccin Immunother* 2022; 18(7): 2090162.

[3] Zhang H, Garcia C, Yu W, et al. National and provincial impact and cost-effectiveness of Haemophilus influenzae type b conjugate vaccine in China: a modeling analysis. *BMC Med* 2021; 19(1): 181.

[4] Feng H, Zhang H, Ma C, Zhang H, Yin D, Fang H. National and provincial burden of varicella disease and cost-effectiveness of childhood varicella vaccination in China from 2019 to 2049: a modelling analysis. *Lancet Reg Health West Pac* 2023; 32: 100639.

[5] National Health and Family Planning Commission of China. China health statistical

yearbook 2020. Beijing: Chinese Academy of Medical Sciences & Peking Union Medical College Press; 2020.

Webappendix 2: Multi-Criteria Decision Analysis (MCDA) of introducing non-NIP vaccines into China's NIP

Multi-Criteria Decision Analysis (MCDA) is a systematic and theory-based approach used to perform comparative evaluations of several competing alternatives, such as healthcare interventions, based on their performance across multiple and often conflicting criteria[1]. The primary purpose of MCDA is to assist decision-makers in making well-informed choices in complex scenarios where trade-offs among criteria are necessary. MCDA offers a more structured, transparent, and comprehensive framework compared to traditional deliberative processes. Stemming from the domain of operations research, MCDA has been extensively utilized in diverse fields, including management, engineering, environmental sciences, etc. Notably, the domain of Health Technology Assessment (HTA) has begun to explore the potential integration of MCDA into its decision-making processes. Prominent national HTA agencies, such as the National Institute for Health and Clinical Excellence (NICE) in the United Kingdom, the Institute for Quality and Efficiency in Health Care (IQWiG) in Germany, and the Canadian Agency for Drugs and Technologies in Health (CADTH), are contemplating the integration of MCDA to bolster their decision-making frameworks[2]. In our analysis, we followed the nine typical steps of the MCDA methodology to investigate the introduction of non-NIP vaccines into China's NIP:

1. Define the Decision Problem. Our decision problem involved determining the prioritization hierarchy for the introduction of four non-NIP childhood vaccines into China's NIP.

2. Identify Alternatives. We listed four childhood vaccines, including pneumococcal conjugate, rotavirus, Hib and varicella vaccines, as the possible alternatives.

3. Determine Criteria. We selected four dimensions for evaluation: disease burden, vaccine coverage, inequalities, and cost-effectiveness. Data for these dimensions were obtained from previously published studies in the literatures, as presented in tables 1 & 2 & 3 in the main manuscript and the following Webappendix table 1. For example, we gained coverage rates and inequalities for four childhood non-NIP vaccines from Zhang et al (2022) [3].

Webappendix table 1. The parameters and data source for each attribute

Criteria	Vaccine (Alternatives)				Weight[4]
	PCV13	Rotavirus (pentavalent)	Hib	Varicella	
Disease burden[a,5,6,7]	7,234	5,855	2,915	0	0.4
Vaccine coverage[b,3]	5.1	1.8	25.0	67.1	0.2
Inequalities[c,3]	0.64	0.77	0.43	0.13	0.2
Cost-effectiveness (ICER, at current market price)[6,7,8,9]	18,628	8,836	7,999	-2,448	0.2
Cost-effectiveness (ICER, at 50% reduced market price)[6,7,8,9]	8,037	1,550	4,447	-18,916	-[d]

The references in the square brackets are listed at the end of Webappendix 2.

a Given that death is the most significant health outcome, we used death as a representation of disease burden.
b Following the WHO's vaccination recommendations and position papers, we adopted a three-dose vaccination rate for PCV13, rotavirus (pentavalent), and Hib vaccines, and a one-dose vaccination rate for varicella vaccine.
c Inequalities were quantified using concentration indices derived from a multidimensional equity model.
d We applied a consistent weight of 0.2 for the sensitivity analysis.

4. Weight the Criteria: We assigned weights to the criteria based on their relative importance in the decision-making process. These weights were derived from a study conducted by the China CDC[4], which gauged their significance in the context of vaccine prioritization for China's NIP. The weights were estimated using a modified Delphi technique (MDT) to develop and refine an indicator system to prioritize vaccines and make policy recommendations concerning their introduction into China's NIP.

5. Score the Alternatives: To make performance measures on various criteria comparable and combinable, we used the Single Attribute Value Function (SAVF) to transform the performance of an alternative on a single criterion (such as disease burden or inequalities in the study) into a comparable value score[10,11]. The SAVF was essential, especially when dealing with criteria that employed different units or scales. The SAVF could be either increasing or decreasing. In our analysis, we adopted an interpretation in which higher scores signified more favorable outcomes. Therefore, we set disease burden and inequalities to be higher for higher scores, and vaccine coverage and ICER to be lower for higher scores. The SAVFs encompassed three types, including exponential, linear, and categorical. We used exponential SAVFs in our analysis differentiated by the bisection technique. The exponential value function for exponential SAVF was defined as follows:

$$V(x) = 1 - e^{-k \times x}$$

Where:

- $V(x)$ is the value of the attribute.
- x is the level of the attribute.
- k is a constant that determines the curvature or sensitivity of the function. Here we set it to 3.

6. Apply Aggregation Method: Once the SAVFs were established for each criterion, we combined the weights and scores to determine the overall value (or utility) of an alternative. This overall value was computed using a Multiple Attribute Value Function (MAVF). The MAVF aggregated the individual criteria values into an overall score, usually taking into account the weights or importance of each criterion. We used the weighted sum model, which was the most commonly used method to calculate the MAVF score. The weighted sum model was an approach that the value for each criterion was multiplied by its weight, and the results were then summed up. The formula was shown as follows:

$$V(A) = \sum_{i=1}^{n} w_i \times v_i(A)$$

Where:

- V(A) is the overall value or score of alternative A.
- n is the number of criteria.
- W_i is the weight of the ith criterion.
- $v_i(A)$ is the value of alternative A in the ith criterion, as determined by the SAVF.

7. Rank and Select: We determined the best alternative based on their aggregated MAVF scores. The non-NIP vaccine with the highest aggregated MAVF score was considered the highest priority for inclusion in China's NIP.

8. Sensitivity Analysis: To assess the robustness of our conclusions, we conducted a sensitivity analysis by adjusting vaccine market prices downward by 50%.

9. Communicating Results and Making Decision: We prioritized the four non-NIP vaccines based on the final aggregated MAVF scores and the results of the sensitivity analysis, aiming at facilitating informed decision-making.

All analyses were done using R statistical software version 4.1.0 (R Project for Statistical Computing) and R package ("DecisionAnalysis").

References

[1] Gongora-Salazar P, Rocks S, Fahr P, et al. The use of multicriteria decision analysis to support decision making in healthcare: an updated systematic literature review. *Value Health* 2023; 26(5): 780-90.

[2] Diaby V, Goeree R. How to use multi-criteria decision analysis methods for reimbursement decision-making in healthcare: a step-by-step guide. *Expert Rev Pharmacoecon Outcomes Res*. 2014 Feb;14(1):81-99.

[3] Zhang H, Lai X, Mak J, at al. Coverage and equity of childhood vaccines in China. *JAMA Netw Open*. 2022 Dec 1;5(12):e2246005.

[4] Ma C, Li J, Wang N, et al. Prioritization of vaccines for inclusion into China's expanded program on immunization: evidence from experts' knowledge and opinions. *Vaccines* 2022; 10(7): 1010.

[5] Lai X, Wahl B, Yu W, et al. National, regional, and provincial disease burden attributed to Streptococcus pneumoniae and Haemophilus influenzae type b in children in China: Modelled estimates for 2010-17. *Lancet Reg Health West Pac* 2022; **22**: 100430.

1.

[6] Wang J, Zhang H, Zhang H, et al. Public health impact and cost-effectiveness of rotavirus vaccination in China: comparison between private market provision and national immunization programs. *Hum Vaccin Immunother* 2022; **18**(7): 2090162.

[7] Feng H, Zhang H, Ma C, et al. National and provincial burden of varicella disease and cost-effectiveness of childhood varicella vaccination in China from 2019 to 2049: a modelling analysis. *Lancet Reg Health West Pac* 2023; **32**: 100639.

[8] Lai X, Garcia C, Wu D, et al. Estimating national, regional and provincial cost-effectiveness of introducing childhood 13-valent pneumococcal conjugate vaccination in China: a modelling analysis. *Lancet Reg Health West Pac* 2023; 32: 100666.

[9] Zhang H, Garcia C, Yu W, et al. National and provincial impact and cost-effectiveness of Haemophilus influenzae type b conjugate vaccine in China: a modeling analysis. *BMC Med* 2021; **19**(1): 181.

[10] Frazão TDC, Camilo DGG, Cabral ELS, et al. Multicriteria decision analysis (MCDA) in health care: a systematic review of the main characteristics and methodological steps. *BMC Med Inform Decis Mak* 2018; 18(1): 90.

[11] Uzun B, Ozsahin I, Agbor VO, et al. Chapter 2 - Theoretical aspects of multi-criteria decision-making (MCDM) methods. In: Ozsahin I, Ozsahin DU, Uzun B, eds. Applications of multi-criteria decision-making theories in healthcare and biomedical engineering: Academic Press; 2021: 3-40.

Webappendix 3: Governmental budget impact analysis of introducing non-NIP vaccines into China's NIP

We conducted a governmental budget impact analysis to explore the financial implications of China's decision to include four non-NIP vaccines in the NIP in 2023. The results were reported in table 5 of the main manuscript. Here we outlined the methodology for conducting the governmental budget impact analysis.

1. We assumed that the newborn cohort in 2023 would resemble that of 2022, as the number of newborns (will be immunized with non-NIP vaccines) is not yet available in China. The number of newborns in 2022 was 9.56 million in China [1].

2. In alignment with the WHO's recommendations, we followed a 3-dose vaccination strategy for PCV13, rotavirus, and Hib vaccines in the NIP [2-4]. For varicella vaccination, we employed a one-dose plus mass catch-up strategy as it was found to be the most cost-effective in China [5,6], and the two-dose strategy incurred higher government costs.

3. The market price per dose of non-NIP vaccines in 2022 was used to estimate the government costs in 2023: USD 68.12 per dose for PCV13, USD 43.10 per dose for pentavalent rotavirus vaccine, USD 11.62 per dose for Hib vaccine, and USD 20.79 per dose for varicella vaccine[7]. We assumed a vaccination rate of 95% for all vaccines. We calculated the total governmental vaccine procurement costs of a single non-NIP vaccine using the following formula:

   Governmental vaccine procurement cost = [Market price per dose] * [Number of doses per child] * [Number of newborn children] *[Assumed vaccine coverage rates]

   As an example, for PCV13, the governmental vaccine procurement cost of PCV13 = USD 68.12 * 3 doses * 9.56 million*95% = USD 1,856.27 million.

   Applying the same formula, we calculated the governmental vaccine procurement costs of pentavalent, Hib, and varicella vaccines as USD 1174.48 million, USD 316.65 million, and USD 188.77 million, respectively. According to the current immunization financing policy in China, the central government will be responsible for all vaccine procurement costs after the introduction of these non-NIP vaccines into China's NIP.

4. The local government at the provincial, prefecture, and county levels will cover the immunization service costs, which amount to USD 3.62 (CNY 25) per dose. We applied a similar formula to calculate the total immunization service costs paid by the local government. For varicella vaccine, we calculated a one-time catch-up cost, comprising vaccine procurement costs of USD 1,165.28 million and immunization service costs of USD 202.90 million.

References

[1] National Bureau of Statistics of the People's Republic of China. The statistical report of

national economy and social development of the People's Republic of China 2022. 2022. http://www.stats.gov.cn/tjsj/zxfb./202302/t20230227_1918980.html (accessed July 19 2023).

[2] WHO. WHO position paper: Pneumococcal conjugate vaccines in infants and children under 5 years of age. 2019. https://www.who.int/publications/i/item/10665-310968 (accessed July 19 2023).

[3] WHO. Rotavirus vaccines: WHO position paper. 2021. https://www.who.int/publications/i/item/WHO-WER9628 (accessed July 19 2023).

[4] WHO. Haemophilus influenzae type b (Hib) vaccination position paper. 2013. https://www.who.int/publications/i/item/who-wer8839-413-426 (accessed July 19 2023).

[5] WHO. Varicella and herpes zoster vaccines: WHO position paper. 2014. https://www.who.int/publications/i/item/who-wer-8925-265-288 (accessed July 19 2023).

[6] Feng H, Zhang H, Ma C, Zhang H, Yin D, Fang H. National and provincial burden of varicella disease and cost-effectiveness of childhood varicella vaccination in China from 2019 to 2049: a modelling analysis. *Lancet Reg Health West Pac* 2023; 32: 100639.

[7] Yaozh. Prices of biological products in China. https://www.yaozh.com/ (assessed on March 15, 2023).

Webappendix 4: The comprehensive list of citations for individual studies from the existing literature

Criteria and index	Vaccines	Citations
Disease burden	PCV13 and Hib	Lai X, Wahl B, Yu W, et al. National, regional, and provincial disease burden attributed to Streptococcus pneumoniae and Haemophilus influenzae type b in children in China: modelled estimates for 2010-17. *Lancet Reg Health West Pac* 2022; 22: 100430.
	PCV13	Lai X, Garcia C, Wu D, et al. Estimating national, regional and provincial cost-effectiveness of introducing childhood 13-valent pneumococcal conjugate vaccination in China: a modelling analysis. *Lancet Reg Health West Pac* 2023; 32: 100666.
	Rotavirus (pentavalent)	Wang J, Zhang H, Zhang H, et al. Public health impact and cost-effectiveness of rotavirus vaccination in China: comparison between private market provision and national immunization programs. *Hum Vaccin Immunother* 2022; 18(7): 2090162.
	Hib	Zhang H, Garcia C, Yu W, et al. National and provincial impact and cost-effectiveness of Haemophilus influenzae type b conjugate vaccine in China: a modeling analysis. *BMC Med* 2021; 19(1): 181.
	Varicella	Feng H, Zhang H, Ma C, et al. National and provincial burden of varicella disease and cost-effectiveness of childhood varicella vaccination in China from 2019 to 2049: a modelling analysis. *Lancet Reg Health West Pac* 2023; 32: 100639.
Vaccine coverage	PCV13, Hib, Rotavirus, Varicella	Zhang H, Lai X, Mak J, et al. Coverage and equity of childhood vaccines in China. *JAMA Netw Open* 2022; 5(12): e2246005.
Inequalities	PCV13, Hib, Rotavirus, Varicella	Zhang H, Lai X, Mak J, et al. Coverage and equity of childhood vaccines in China. *JAMA Netw Open* 2022; 5(12): e2246005.
Cost-effectiveness (ICER)	PCV13	Lai X, Garcia C, Wu D, et al. Estimating national, regional and provincial cost-effectiveness of introducing childhood 13-valent pneumococcal conjugate vaccination in China: a modelling analysis. *Lancet Reg Health West Pac* 2023; 32: 100666.
	Rotavirus (pentavalent)	Wang J, Zhang H, Zhang H, et al. Public health impact and cost-effectiveness of rotavirus vaccination in China: comparison between private market provision and national immunization programs. *Hum Vaccin Immunother* 2022; 18(7): 2090162.

	Hib	Zhang H, Garcia C, Yu W, et al. National and provincial impact and cost-effectiveness of Haemophilus influenzae type b conjugate vaccine in China: a modeling analysis. *BMC Med* 2021; 19(1): 181.
	Varicella	Feng H, Zhang H, Ma C, et al. National and provincial burden of varicella disease and cost-effectiveness of childhood varicella vaccination in China from 2019 to 2049: a modelling analysis. *Lancet Reg Health West Pac* 2023; 32: 100639.
MCDA weight	-	Ma C, Li J, Wang N, et al. Prioritization of vaccines for inclusion into China's expanded program on immunization: evidence from experts' knowledge and opinions. *Vaccines* 2022; 10(7) :1010.
Immunization service costs	PCV13, Hib, Rotavirus, Varicella	Yu W, Lu M, Wang H, et al. Routine immunization services costs and financing in China, 2015. *Vaccine* 2018; **36**(21): 3041-7.
Vaccine prices	PCV13, Hib, Rotavirus, Varicella	Zhang H, Patenaude B, Ma C, et al. Vaccine pricing strategies in China. *BMJ Glob Health* 2023; 8(7):e011405.
		Yaozhi Website. Prices of biological products. 2023. https://www.yaozh.com/ (accessed Jul 19, 2023).
		State Council of the People's Republic of China. Central government procurement 2022. 2022. http://zycg.cn/freecms/site/zygjjgzfcgzx/cggg/index.html (accessed Jul 19, 2023).
Number of live births and children	-	National Health Commission of the People's Republic of China. China Health statistical yearbook 2020. Beijing: Chinese Academy of Medical Sciences & Peking Union Medical College Press; 2020.
		National Bureau of Statistics of the People's Republic of China. China statistical yearbook 2022. Beijing: China Statistics Press; 2022.
Neonatal, infant and under-five mortality rates	-	National Health Commission of the People's Republic of China. China health statistical yearbook 2020. Beijing: Chinese Academy of Medical Sciences & Peking Union Medical College Press; 2020.
